# The Relationship between the Brexit Vote and Individual Predictors of Prejudice: Collective Narcissism, Right Wing Authoritarianism, Social Dominance Orientation

**DOI:** 10.3389/fpsyg.2017.02023

**Published:** 2017-11-27

**Authors:** Agnieszka Golec de Zavala, Rita Guerra, Cláudia Simão

**Affiliations:** ^1^Goldsmiths, University of London, London, United Kingdom; ^2^Instituto Universitário de Lisboa (ISCTE-IUL), Cis-IUL, Lisbon, Portugal; ^3^Department of Psychology, University of Social Sciences and Humanities, Poznan, Poland; ^4^Católica Lisbon School of Business and Economics, Universidade Católica Portuguesa, Lisbon, Portugal

**Keywords:** Brexit vote, immigration threat, collective narcissism, right wing authoritarianism, social dominance orientation

## Abstract

The Leave campaign in the U.K., which advocated exiting the European Union, emphasized anxiety over immigration and the need to take control of the U.K.'s borders. Citizens who expressed concerns about immigration to the U.K. were more likely to vote to leave. Two correlational studies examined the previously unexplored question of whether the Brexit vote and support for the outcome of the E.U. referendum were linked to individual predictors of prejudice toward foreigners: British collective narcissism (a belief in national greatness), right wing authoritarianism, and social dominance orientation. The results converged to indicate that all three variables were independently related to the perceived threat of immigrants and, via this variable, to the Brexit vote and a support for the outcome of the E.U. referendum. These variables explained the variance in the perceived threat of immigrants and support for the Brexit vote over and above other previously examined predictors such as age, education, or ethnicity, as well as, national identification and national attachment.

## Introduction

On June 23, 2016 almost 52% of British citizens who participated in the European Union (E.U.) referendum in the United Kingdom voted to leave. Individual level explanations that followed this largely unexpected result pointed to the role of voter's age and ethnicity, their cost-benefit and risk calculations, susceptibility to influence political elites and low feelings of attachment to the wider international community (Clarke et al., 2016). Several explanations suggested that the Leave campaign might have mobilized xenophobic attitudes by emphasizing fear of foreigners and the need to take control of the U.K.'s borders to limit free movement of E.U. nationals. In this vein, survey results suggested that those respondents who expressed concerns about immigration to the U.K. were more likely to vote Leave (Hobolt, 2016).

Was the Brexit vote related to xenophobia? To answer this question the present studies examined whether the Brexit vote and support for the referendum's outcome were linked to feeling threatened by immigrants and foreigners and whether they were more likely among people already prone to prejudice. More specifically, we examined whether collective narcissist (i.e., individuals who believe in their nation's unparalleled greatness, Golec de Zavala et al., 2009), authoritarians (those who obey authority and social conventions and reject dissenters, Altemeyer, 1988) and people high in social dominance orientation (those who want to maintain group based hierarchies, Pratto et al., 1994) were more likely to vote to leave the E.U. because they felt threatened by immigrants.

Although, right wing authoritarianism and social dominance orientation have been already implicated in voting for radical right wing parties because of the perceived threat of immigrants (Aichholzer and Zandonella, 2016), collective narcissism has almost never been examined in the context of political behaviors such as voting. However, multiple studies indicate that collective narcissism predicts rejection of outgroups more systematically than national identification (i.e., considering one's own national identity as important and central to the self) or national attachment (i.e., feeling love for and pride of one's own nation) (Mummendey et al., 2001; Herrmann et al., 2009; see also Roccas et al., 2006). Moreover, although research demonstrated that collective narcissism predicts rejection of outgroups independently of right wing authoritarianism and social dominance orientation (for review see, Golec de Zavala, in press), no previous studies examined whether the three variables explain attitudes toward the superordinate group that would undermine the strength of the boundaries between the national ingroup and outgroups, i.e., the European Union.

## Collective narcissism and perceived immigration threat

Previous studies have linked British national identification and attachment to unfavorable attitudes toward immigration to the U.K. and toward the E.U. (Carrey, 2002; Cinnirella and Hamilton, 2007). However, a closer examination of these relationships is desirable in light of the findings that the average association between ingroup identification (Pehrson et al., 2009) or positive ingroup attachment (Leach et al., 2008) and rejection of outgroups is close to zero (Rubin and Hewstone, 1998). Instead, psychological research has differentiated between positive national attitudes that are more likely to be linked to outgroup derogation (e.g., blind or conventional patriotism) and those that are not (e.g., constructive patriotism, national ingroup satisfaction, Kosterman and Feshbach, 1989; Schatz et al., 1999; Leach et al., 2008; Sekerdej and Roccas, 2016).

Collective narcissism is a form of a positive ingroup attitude that, when applied to a nation, is systematically related to rejection of foreigners and national and ethnic minorities (Cai and Gries, 2013; Lyons et al., 2013; Cichocka, 2016; Golec de Zavala, in press). People who score high on the Collective Narcissism Scale (i.e., collective narcissists) endorse the view that their nation's importance and true worth is not sufficiently recognized by others, concur that their nation “deserves special treatment,” and expect that their nation receives its due acknowledgement and respect (Golec de Zavala et al., 2009; Golec de Zavala, in press). Collective narcissism is related to direct and indirect retaliatory hostility in response to real or imagined offenses to the ingroup (Golec de Zavala et al., 2013a, 2016). Collective narcissistic prejudice and rejection of outgroups is driven by a belief that the targeted outgroups threatened the ingroup's safety (Golec de Zavala et al., 2009; Golec de Zavala and Cichocka, 2012) or its narrowly defined “purity” (Gries et al., 2008).

Collective narcissism explains the rejection of outgroups even after controlling for levels of national identification or national attachment. After their links with collective narcissism are controlled for, national identification and national attachment cease to explain outgroup rejection in the context of intergroup threat (with the exception of one study showing an independent relation between outgroup rejection and blind patriotism mediated by the perceived threat after controlling for collective narcissism, Golec de Zavala et al., 2013b, 2016). Given such findings, we expected that collective narcissism might predict political behaviors such as support for anti-immigrant policies, voting for political parties that support such politics or, as in the case of the E.U. referendum, choosing to leave the E.U. However, collective narcissism has not been frequently examined in the context of political behaviors, despite the above evidence suggesting that it may predict voting at least as much as right wing authoritarianism and social dominance orientation whose implications for political behaviors were studied more frequently.

One of the few studies that examined the implications of collective narcissism in the context of voting showed that collective narcissism predicted voting for Donald Trump in 2016 Presidential election in the U.S. independently of demographic variables, authoritarianism, and national identification. Thus, collective narcissism predicted support for the political candidate whose campaign emphasized alleged threat to the country's unique and privileged position (“America first” or “Make America great a*gain*”) and who advocated anti-immigrant policies (Federico and Golec de Zavala, 2017). Such findings suggest that collective narcissists may be mobilized by political rhetoric emphasizing threatened national uniqueness. Since the Leave campaign heavily relied on such rhetoric, we expected that British collective narcissism would predict the Brexit vote via the perception of immigrants in the UK as threatening.

There are other reasons to expect that collective narcissists may perceive immigrants as threatening. First, immigrants' foreign values and customs may undermine the “purity” of British identity (Gries et al., 2008). In addition, misunderstandings that are frequent in interactions between nationals of different countries may seem particularly aggravating to collective narcissists. Collective narcissism has been linked to a tendency to perceive ambiguous intergroup situations as intentional offenses to the ingroup, to which collective narcissists react aggressively (Golec de Zavala et al., 2016). Immigrants and foreign workers who fair better than host country nationals pose a threat of unfavorable intergroup comparisons that may undermine perceived superiority of the host group (Esses et al., 2013; Murray and Marx, 2013). Such comparisons are aversive to collective narcissists sensitivity to threats to the ingroup's image. Collective narcissism predicts hostile responses to such perceived threats (Golec de Zavala, in press). Finally, the expectation that collective narcissism predicts the perceived threat of immigrants is in line with the Intergroup Emotions Theory which posits that group-based emotions arise when people appraise the implications of intergroup situations in terms of their implication for the ingroup (Smith and Mackie, 2015). The results reviewed above indicate that collective narcissists are likely to appraise immigrants as a threat to their ingroup and react to them with negative, hostile, emotions. Such group-based hostility might have been expressed in the Brexit vote. A post-referendum increase in the narrative about national uniqueness and superiority, on the one hand, and in hate speech and discrimination against foreigners in the U.K., on the other hand, offered anecdotal support for this expectation (Forster, 2016).

## Collective narcissism, right wing authoritarianism, social dominance orientation and prejudice

Apart from the threat to the national ingroup image, the Leave campaign brought up other issues that might have linked the perceived threat of immigration to the U.K. with two correlates of collective narcissism and robust predictors of prejudice: right wing authoritarianism (Altemeyer, 1988) and social dominance orientation (Pratto et al., 1994; for their links to prejudice see, Whitley, 1999; Ekehammar et al., 2004; Dru, 2007; Duckitt and Sibley, 2007; Asbrock et al., 2010).

Research indicated that the links between right wing authoritarianism and social dominance orientation and prejudice are additive rather than interactive: the two variables predict prejudice independently and for different reasons (Ekehammar et al., 2004; Sibley et al., 2007a,b). Right wing authoritarianism, which has been indirectly implicated in the Brexit vote (Kaufman, 2016), is a three-faceted attitude syndrome combining submission to strength-based authority, aggressiveness toward social deviants and dissenters, support for conventionalism, order and a non-diverse environment (Altemeyer, 1988). It is related to a belief that the world is a dangerous place. Thus, right wing authoritarianism is related to prejudice toward those social groups that appear to threaten the traditional status quo (Duckitt, 2006; Duckitt and Sibley, 2007).

In this vein, research has linked right wing authoritarianism with hostile responses to social threats (Lavine et al., 1999; Hibbing et al., 2014), political conservatism (Jost et al., 2003; Duckitt et al., 2010), and more specifically, to voting for right wing political parties and candidates advocating rejection of immigrants in Europe (Aichholzer and Zandonella, 2016), in the U.S. (Crawford et al., 2013; MacWilliams, 2016; Choma and Hanoch, 2017; Crowson and Brandes, 2017), or in Latin America (Cohen and Smith, 2016). The Leave campaign emphasized the economic threat that immigrants pose to British nationals. In addition, it framed the principles pursued by the E.U. as not complying with traditional British values. Such framing might have mobilized people high in right wing authoritarianism to reject immigrants and to vote to leave the E.U. in order to protect the traditional social order.

Social dominance orientation refers to individual degree of preference for group-based hierarchies (Pratto et al., 1994). It is related to the belief that the world is a “competitive jungle,” where groups need to fight for superiority. Thus, social dominance orientation is related to prejudice toward groups threatening the ingroup's status (Duckitt, 2006). Indeed, previous studies have linked social dominance orientation with anti-egalitarian, anti-immigrant attitudes (Sidanius et al., 2000; Kteily et al., 2017), political conservatism (Ho et al., 2012), and voting for radical, right wing political parties advocating maintenance of group-based social hierarchies (Cornelis and Van Hiel, 2015; Aichholzer and Zandonella, 2016). The Leave campaign alluded to the U.K.'s subsidiary role in the E.U. (Elliott, 2016). Such allusions might have mobilized people high in social dominance to vote to leave the E.U. to compete for higher international status. Individuals high in social dominance orientation might also have been mobilized to reject immigrants who fare well in the U.K., thus posing a status threat to British nationals.

Thus, it is likely that the Brexit vote was an expression of hostility toward immigrants in response to different concerns that immigration raised for collective narcissists, authoritarians, and people high in social dominance orientation. More specifically, the perceived threat of immigrants was likely to be motivated by the collective narcissistic concern regarding threaten national uniqueness, authoritarian concern about maintaining the normative status quo, and the concern regarding the protection of the elevated international status related to social dominance orientation.

## The present studies

The present studies tested the hypothesis that collective narcissism, right wing authoritarianism and social dominance orientation *independently* predicted the Brexit vote and a support for the outcome of the E.U. referendum because they *independently* predicted the perceived threat of immigrants. As such, these are the first studies to compare the relationships of the three robust predictors of prejudice with attitudes toward immigrants and the E.U. in the U.K. We hypothesized that collective narcissism, right wing authoritarianism and social dominance orientation remain related to the perceived threat of immigrants and the Brexit vote after participants' age, gender, education, ethnicity, reported national group and national ingroup identification (Study 1) and national attachment (Study 2) are accounted for. Previous studies indicate that neither ingroup identification nor ingroup attachment is positively related to outgroup rejection after collective narcissism is controlled for (Golec de Zavala et al., 2016). Thus, Study 1 looked at the role of collective narcissism as compared to national identification and Study 2 compared collective narcissism and national attachment as predictors of the perceived threat of immigrants, the referendum vote and support for the referendum's outcome.

## Study 1

Study 1 was conducted in July 2016 just after the E.U. referendum in the U.K. It tested whether collective narcissism, right wing authoritarianism and social dominance orientation were related to the Brexit vote and a support for the referendum's outcome because of their unique relationships with the perceived threat of immigrants. Study 1 also tested whether the link between collective narcissism and the Brexit vote was specific to collective narcissism and not explained by the strength of national identification.

### Participants

Study 1 was conducted online online using the Qualtrics platform via the Prolific Academic research panel (www.prolific.ac) among 280 British citizens who voted in the European referendum, of whom 194 reported voting to remain. We obtained a sample of 285 participants but 5 participants did not meet the participation criterion (having voted in the referendum). Their data was not taken into account in the analyses. The mean age of the remaining 280 participants was 34.58 (*SD* = 12.94), 184 of the respondents were women. One hundred and fifty-one participants reported their nationality as English, 2 as Scottish, 15 Welsh, 90 British, 17 U.K., 5 as Other. The sample size was set to be over 250 participants based on research suggesting that correlations stabilize at more or less this number of participants (Schönbrodt and Perugini, 2013). All participants were paid a small fee in exchange for their participation.

### Procedure

Participants were told that the study examined attitudes toward the E.U. in relation to national attitudes and personality. After reading the informed consent form and consenting by clicking the “By clicking here you give your consent to participate in this study” button, participants could proceed to the study. They were first asked to provide their demographic data. Next, they responded to individual difference measurements presented in random order to each participant. The order of items was also randomized. Then, participants were thanked, paid, and debriefed. In both studies data collection ceased on a predetermined date and data were not observed prior to analyses.

#### Measurements

Study 1 controlled for participants' age, gender (0 = “*Male,”* 1 = “*Female”*), education, ethnicity (0 = “*Black”* or “*Asian”* or “*Other,”* 1 = “*White”*) and reported national group (0 = “*English”* or “*Welsh”* or “*Scottish”* or “*Irish*,” 1 = “*British”* or “*U.K.”*).

##### Collective narcissism

Collective narcissism was measured using the 5-item Collective Narcissism Scale (Golec de Zavala et al., 2009, 2013b, i.e., “*My national group deserves special treatment,” “*I will never be satisfied until my national group gets all it deserves,” “It really makes me angry when others criticize my national group,” “If my national group had a major say in the world, the world would be a much better place,” “Not many people seem to fully understand the importance of my national group,” α = 0.87). Items were answered on scales from “1” = “*I strongly disagree”* to “6” = “*I strongly agree.”*

##### Right wing authoritarianism

Right wing authoritarianism was measured by a 10-item version of the Right-Wing Authoritarianism Scale (Zakrisson, 2005, e.g., “*Obedience and respect for authority are the most important virtues children should learn,”* α = 0.78). Items were answered on scales from “1” = “*I strongly disagree”* to “7” = “*I strongly agree.”*

##### Social-dominance orientation

Social-dominance orientation was measured by a 4-item version of the Social Dominance Orientation Scale (Pratto et al., 2013, e.g., “*We should not push for group equality*,” reversely coded, α = 0.82). Items were answered on scales from “1” = “*I strongly disagree”* to “7” = “*I strongly agree.”*

##### National identification

National identification was measured by the Centrality subscale of the Social Identity Scale proposed by Cameron (2004, e.g., “*Being from my national group is an important reflection of what I am.,”* “Being from my national group is not important for how I think of what type of person I am.,” “I have a strong feeling of belonging to my national group.,” “In general, being from my national group has little to do with the way I feel about myself.,” “Being from my national group is not a central factor of my social relations,” “In general, being from my national group is an important part of my self-image.,” α = 0.87). Items were answered on scales from “1” = “*I strongly disagree”* to “7” = “*I strongly agree.”*

##### Perceived threat of immigrants in the U.K.

Perceived threat of immigrants in the U.K. was measured by 10 items asking to what extent immigrants pose a realistic and symbolic threat to the U.K. Specifically, participants were asked to what extent they agreed that immigrants and foreign workers “*threaten our jobs and economic opportunities”; “threaten our personal possessions”; “threaten our personal rights and freedoms”; “violate reciprocity relations by choice”; “threaten our social coordination and functioning”; “violate our trust”; “threaten our physical health”; “hold values inconsistent with those of my national group”; “endanger our physical safety”; “violate reciprocity relations because of a lack of ability”* (Cottrell and Neuberg, 2005, α = 0.95). Items were answered on scales from “1” = “*I strongly disagree”* to “7” = “*I strongly agree.”* The principal component factor analysis resulted in one factor solution with factor loading of 8.16. Items loadings ranged from 0.66 to 92. Thus, the items were averaged to form one index of the perceived threat of immigrants to the U.K.

##### Brexit vote

Brexit vote was measured by asking participants how they voted in the E.U. referendum: “*How did you vote in the European referendum on 26th of June?”* (0 = “*Remain”*; 1 = “*Leave”*).

##### Support for the referendum's outcome

Support for the referendum's outcome was measured by the question: “*To what extent do you feel … about the outcome of the European referendum?”* Following emotions were assessed: “*happy”; “disappointed”; “proud”; “shocked”; “frightened”; “disgusted”; “unhappy”; “thrilled”;* and “*worried.”* Positive and reversed negative emotion formed a reliable scale, α = 0.94. Items were answered on scales from “1” = “*Not at all”* to “5” = “*Very much so.”*

### Results

#### Predictors of prejudice and the referendum vote via the perceived threat of immigrants

The results in Table 1 show that all continuous variables in Study 1 were positively correlated. In order to test the hypothesis that collective narcissism, right wing authoritarianism and social dominance orientation were related to the referendum vote because they were associated with the perceived threat of immigrants, we first performed multiple mediation analyses in a multiple regression context with the self-reported vote in the E.U. referendum as a binary outcome variable using PROCESS (Model 4, Hayes, 2013). The macro estimates the direct and indirect effects, as well as the path from the proposed mediator to the outcome using logistic regression. Logistic regression coefficients are estimated using a Newton–Raphson iteration algorithm. Regression path coefficients for the dichotomous outcome are the maximum-likelihood-based logistic regression coefficients or the log odds ratios. The confidence intervals are associated with the Wald test (*Z*-value).

**Table 1:** Descriptive statistics and correlations for the measurements of Study 1, *N* = 280.

**Variables**	**Mean**	***SD***	**Correlations**				
			**1**.	**2**.	**3**.	**4**.	**5**.
1. Collective narcissism	2.62	1.14	—				
2. National identity	3.64	1.28	0.44^***^	—			
3. Social dominance orientation	2.21	1.08	0.32^***^	0.24^***^	—		
4. Right-wing authoritarianism	3.03	0.98	0.58^***^	0.39^***^	0.38^***^	—	
5. Perceived threat of immigrants	2.32	1.28	0.60^***^	0.33^***^	0.52^***^	0.59^***^	—
6. Support for the outcome	3.70	1.18	0.50^***^	0.30^***^	0.34^***^	0.45^***^	0.65^***^

*** *p < 0.001*.

**Figure 1:**
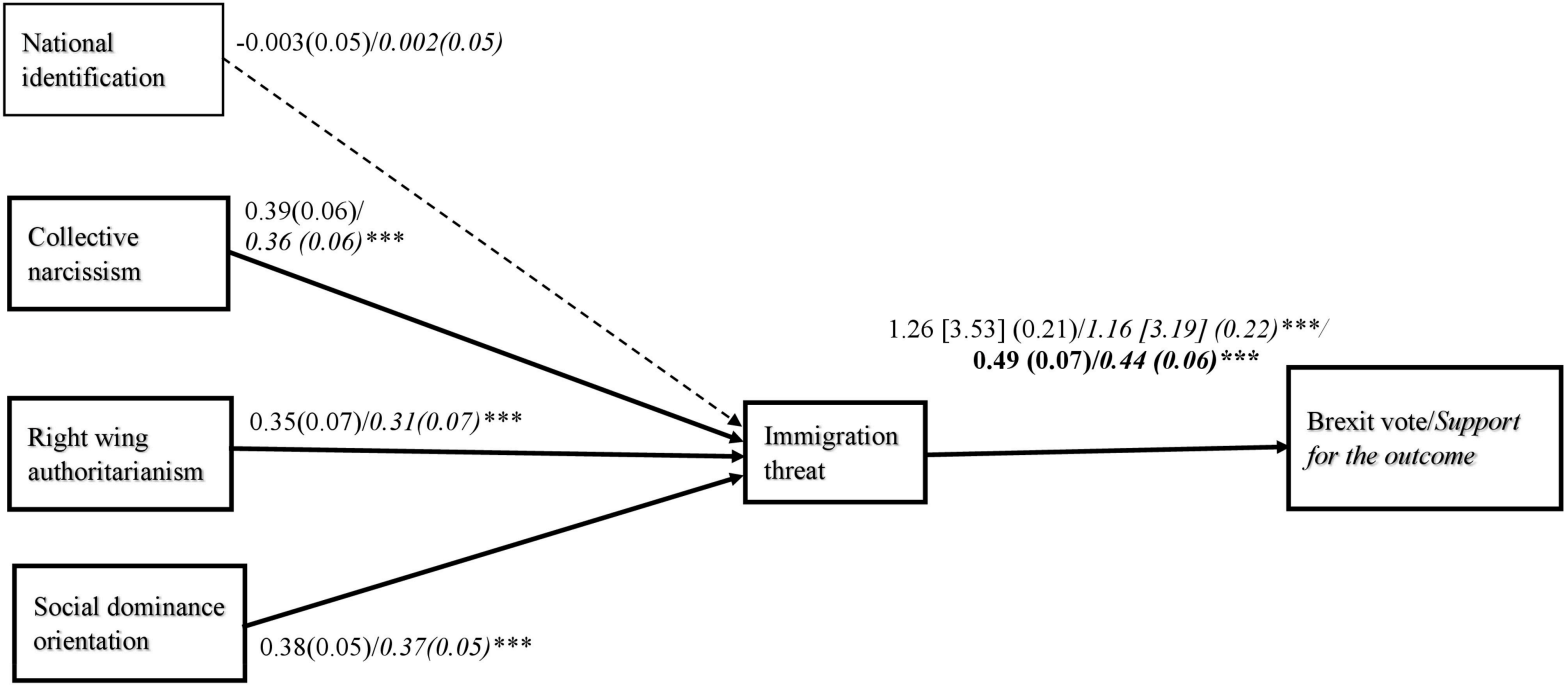
Relationship between variables in Study 1. Coefficients for the binary outcome variable represent log odds [odd ratios] and standard errors are in parentheses. Coefficients for the continuous variables represent unstandardized regression weights. Numerals in italic font correspond to analyses with covariates (national identification, age, gender, education, ethnicity, and indicated national group). Numerals in bold font correspond to unstandardized regression weights and standard error in analyses predicting the continuous support for the outcome of the referendum. ^***^p < 0.001.

In order to estimate the direct and indirect effects of all variables we ran the multiple mediation analysis three times, each time with a different variable as a predictor in the model (a model for each predictor: collective narcissism, right wing authoritarianism and social dominance orientation), the perceived threat of immigrants as a mediator, the referendum vote as the outcome variable and the remaining variables as covariates. Mathematically, all resulting paths, direct and indirect effects, are the same as if they were estimated simultaneously (Hayes, 2013) (Table 2, Figure 1). This analysis allowed us to test the hypothesis that collective narcissism, right wing authoritarianism, and social dominance orientation independently predict the Brexit vote via the perception of immigrants as threatening. We ran the analyses without demographic covariates first. Next, the analyses controlled for participants' age, gender, education, ethnicity, and national identification in order to demonstrate that collective narcissism, right wing authoritarianism and social dominance orientation accounted for a unique portion of variance in the referendum vote. Finally, in order to compare the relative contribution of collective narcissism and national identification to explaining the variance in the perceived threat of immigrants and the Brexit vote, we performed the same multiple mediation analysis with national identification as a predictor instead of collective narcissism, first without controlling for the overlap between the two variables (but controlling for the overlap with right wing authoritarianism and social dominance orientation), next entering collective narcissism as a covariate, and finally including all covariates (results of the latter analysis are presented in Table 2, Figure 1). All predictors were mean centered prior to the analyses. We used the default bootstrapping with 10,000 samples in all analyses to construct the confidence intervals for the observed effects.

**Table 2:** Direct and indirect effects of all variables on the Brexit vote and support for the referendum's outcome, Study 1 and 2.

	**Direct effect**			**Indirect effect**				
	**Coefficient**	**SE**	***p***	**Coefficient**	**SE**	**95% CI**	***Z***	***p***
**STUDY 1, BREXIT VOTE (WITHOUT COVARIATES)**								
Collective narcissism	0.33	0.20	0.10	0.49	0.13	[0.28; 0.75]	4.40	<0.001
Right wing authoritarianism	0.07	0.24	0.76	0.45	0.15	[0.21; 0.77]	3.87	<0.001
Social dominance orientation	−0.17	0.19	0.36	0.47	0.14	[0.25; 0.77]	4.54	<0.001
National identity	0.13	0.16	0.41	−0.004	0.06	[−0.13; 0.11]	−0.06	0.95
**STUDY 1, BREXIT VOTE (WITH COVARIATES)**								
Collective narcissism	0.37	0.22	0.09	0.42	0.13	[0.21; 0.68]	3.96	<0.001
Right wing authoritarianism	−0.02	0.25	0.95	0.36	0.14	[0.15; 0.68]	3.39	<0.001
Social dominance orientation	−0.11	0.20	0.56	0.43	0.14	[0.21; 0.73]	4.18	<0.001
National identity	0.15	0.17	0.37	0.002	0.06	[−0.12; 0.11]	0.03	0.97
**STUDY 1, SUPPORT FOR THE REFERENDUM'S OUTCOME (WITHOUT COVARIATES)**								
Collective narcissism	0.15	0.06	0.03	0.19	0.04	[0.12; 0.28]	5.04	<0.001
Right wing authoritarianism	0.04	0.07	0.54	0.17	0.05	[0.09; 0.28]	4.29	<0.001
Social dominance orientation	−0.02	0.06	0.79	0.18	0.04	[0.11; 0.28]	5.26	<0.001
National identity	0.05	0.05	0.29	−0.002	0.02	[−0.05; 0.04]	−0.06	0.95
**STUDY 1, SUPPORT FOR THE REFERENDUM'S OUTCOME (WITH COVARIATES)**								
Collective narcissism	0.13	0.06	0.05	0.16	0.04	[0.10; 0.25]	4.62	<0.001
Right wing authoritarianism	0.04	0.08	0.09	0.14	0.04	[0.07; 0.23]	3.79	<0.001
Social dominance orientation	−0.03	0.06	0.64	0.16	0.04	[0.10; 0.25]	4.98	<0.001
National identity	0.06	0.05	0.23	0.001	0.02	[−0.04; 0.04]	0.03	0.97
**STUDY 2, BREXIT VOTE (WITHOUT COVARIATES)**								
Collective narcissism	0.12	0.22	0.58	0.42	0.13	[0.20; 0.70]	3.69	0.002
Right wing authoritarianism	0.21	0.23	0.36	0.26	0.12	[0.07; 0.52]	2.59	0.010
Social dominance orientation	−0.08	0.20	0.69	0.28	0.11	[0.10; 0.51]	2.99	0.003
National attachment	−0.001	0.18	1.00	0.08	0.07	[−0.04; 0.21]	1.16	0.24
**STUDY 2, BREXIT VOTE (WITH COVARIATES)**								
Collective narcissism	0.13	0.22	0.56	0.38	0.13	[0.17; 0.65]	3.53	0.004
Right wing authoritarianism	0.17	0.24	0.49	0.19	0.12	[−0.004; 0.45]	2.05	0.04
Social dominance orientation	−0.08	0.21	0.72	0.28	0.11	[0.11; 0.51]	2.98	0.003
National attachment	0.003	0.19	0.99	0.07	0.07	[−0.05; 0.21]	1.15	0.25
**STUDY 2, SUPPORT FOR THE REFERENDUM'S OUTCOME (WITHOUT COVARIATES)**								
Collective narcissism	−0.03	0.08	0.68	0.22	0.05	[0.12; 0.33]	4.42	<0.001
Right wing authoritarianism	0.07	0.08	0.40	0.14	0.06	[0.04; 0.26]	2.83	0.005
Social dominance orientation	0.05	0.07	0.46	0.15	0.05	[0.06; 0.25]	3.36	<0.001
National attachment	0.03	0.06	0.56	0.04	0.03	[−0.02; 0.10]	1.19	0.23
**STUDY 2, SUPPORT FOR THE REFERENDUM'S OUTCOME (WITH COVARIATES)**								
Collective narcissism	−0.03	0.08	0.71	0.20	0.05	[0.11; 0.31]	4.23	<0.001
Right wing authoritarianism	0.05	0.08	0.58	0.10	0.05	[0.06; 0.21]	2.18	0.03
Social dominance orientation	0.04	0.07	0.56	0.15	0.05	[0.06; 0.25]	3.38	<0.001
National attachment	0.04	0.06	0.47	0.04	0.03	[−0.02; 0.10]	1.18	0.24

*The coefficients for the binary outcome (the referendum vote) are the log odds ratios. The coefficients for the continuous outcome (support for the referendum outcome) are unstandardized multiple regression weights*.

The model we tested first, with collective narcissism, right wing authoritarianism, and social dominance orientation independently predicting the perceived threat of immigrants and the Brexit vote, was significant, *R*_*CS*_^2^ = 0.35 (0.50 Nagelkerke), $\chi_{\left(1\right)}^{2}$ = 223.59, *p* < 0.001. Collective narcissism, right wing authoritarianism and social dominance orientation were independently, positively related to the perceived threat of immigrants. The indirect relationships between collective narcissism, right wing authoritarianism and social dominance orientation via the perceived threat of immigrants were significant. Adding covariates to the analyses did not change the pattern of the results (Table 2, Figure 1).

In order to assess the role of national identification, we first ran the mediation model with national identification as a predictor instead of collective narcissism (adding right wing authoritarianism and social dominance orientation as covariates). The whole model was significant, *R*_*CS*_^2^ = 0.35 (0.49 Nagelkerke), $\chi_{\left(1\right)}^{2}$ = 225.64, *p* < 0.001. However, the direct effect and indirect effects of national identification were not significant, *b* = 0.20, SE = 0.15, *z* = 1.29, *p* = 0.20, 95%CI [−0.10; 0.50] and IE = 0.11, SE = 0.07, 95%CI [−0.01; 0.25], *z* = 1.61, *p* = 0.11, respectively. When collective narcissism and other covariates were entered into the equation the direct and indirect effects of national identification were reduced but the overall pattern of relationships remained unchanged (Table 2).

#### Predictors of prejudice and support for the referendum's outcome via the perceived threat of immigrants

Next, we performed the same multiple mediation analysis using support for the referendum outcome as a continuous outcome variable. Collective narcissism, right wing authoritarianism, and social dominance orientation were entered as predictors, the perceived threat of immigrants as the mediator and a support for the referendum's outcome as the outcome variable. The analyses were performed three times using PROCESS (Model 4, Hayes, 2013). Next, the analyses were performed with national identification and demographic variables as covariates. Finally, the analyses were performed with national identification as a predictor without, and then with collective narcissism as a covariate.

The first analysis showed that collective narcissism, right wing authoritarianism and social dominance orientation independently indirectly predicted support for the outcome of the referendum via the perceived threat of immigrants which explained variance in, *R*^2^ = 0.44, *F*_(4, 275)_ = 54.26, *p* < 0.001. Analyses with covariates did not change the pattern of the relationships (Table 2, Figure 1). The analyses with national identification entered as a predictor without controlling for its overlap with collective narcissism produced a significant model, *R*^2^ = 0.43, *F*_(4, 275)_ = 52.54, *p* < 0.001. The direct effect and indirect effects of national identification were not significant, *b* = 0.08, SE = 0.05, *t*_(275)_ = 1.68, *p* = 0.09, 95%CI [−0.01; 0.17] and IE = 0.04, SE = 0.02, 95%CI [−0.003; 0.09], *z* = 1.64, *p* = 0.10, respectively. When collective narcissism was entered as a covariate, the direct and indirect effects of national identification were reduced to values presented in Table 2. Especially, the indirect effect was reduced from marginally significant to non-significant. This pattern of results remained unchanged when demographic covariates were also controlled.

#### Relative importance of all predictors of prejudice for the perceived threat of immigrants and support for the referendum's outcome

Finally, because the analyzed predictors of perceived threat of immigrants and support for the referendum's outcome were correlated, relative importance indices were computed to assess the unique contribution of each predictor in the context of possible multicollinearity: dominance weights, relative importance weights, and incremental *R*^2^ (Braun and Oswald, 2011). Those indices determine the unique and combined contribution of collective narcissism, right wing authoritarianism, social dominance orientation, and national identification to explaining variance in the perceived threat of immigrants and support for the referendum's outcome.

Regression weights of strongly correlated predictors may not give an adequate indicator of the unique contribution of each variable because they change with covariance relationships, and therefore may be sample-specific and not easily generalizable. Dominance weights give a more accurate assessment of the hierarchy of importance of the correlated predictors. General dominance weights are computed by averaging the given predictor's incremental validity across all possible submodels that involve that predictor. This analysis reduces the importance of redundant predictors when multicollinearity is present. Relative importance weights indicate the proportionate contribution of each predictor to the variance explained in the outcome variable. Incremental *R*^2^ analysis reflects the unique contribution of each predictor after the variance accounted for by the remaining predictors has been partialled out of the outcome. It represents the increase in *R*^2^ when the predictor is entered last in a stepwise fashion, indicating the unique impact of that predictor in the model (For detailed description of the computations of each weights please see Braun and Oswald, 2011).

The relative importance indices converged to indicate that collective narcissism, right wing authoritarianism and social dominance orientation independently contributed to explaining the variance in the perceived threat of immigrants, in this order of importance. In comparison, the national identification contribution was decidedly minor (Table 3). In the event of a support for the referendum's outcome, collective narcissism, and right wing authoritarianism were the main predictors, whereas the contribution of social dominance orientation was smaller and the contribution of national identification was negligible.

**Table 3:** Comparison of relative importance of all variables in explaining variance in the perceived threat of immigrants and in support for the referendum's outcome, Study 1, *N* = 280.

**Variables**	**VIF**	**Study 1 (*perceived threat of immigrants*, Overall *R*^2^ = 0.53)**				**Study 1 (*support for the outcome*, Overall *R*^2^ = 0.31)**			
		**Regression weights**	**Dominance weights**	**Relative importance weights**	**Incremental *R*^2^**	**Regression weights**	**Dominance weights**	**Relative importance weights**	**Incremental *R*^2^**
Collective narcissism	1.65	0.34	0.18	0.18	0.07	0.32	0.14	0.13	0.06
National identification	1.29	−0.002	0.03	0.03	0.00	0.05	0.03	0.03	0.002
Social dominance orientation	1.19	0.31	0.15	0.15	0.08	0.15	0.05	0.06	0.02
Right wing authoritarianism	1.65	0.28	0.16	0.16	0.06	0.19	0.09	0.09	0.02

Together, the results of Study 1 strongly suggest that collective narcissism, right wing authoritarianism and social dominance orientation were related to the perceived threat of immigrants, and indirectly predicted the Brexit vote and support for the referendum's outcome. National identification did not predict the perceived threat of immigrants, the Brexit vote or support for referendum's outcome after its overlap with collective narcissism, right wing authoritarianism and social dominance orientation was controlled for. We sought to replicate those results to examine whether they generalize to a different sample of British referendum voters. Given the fact that all main predictors were significantly correlated, repeated pattern of relationships in Study 2 would increase our confidence in the replicability of our results. In addition, in Study 2 the role of collective narcissism in predicting the Brexit vote was compared to the role of national attachment.

## Study 2

Study 2 was conducted just after the U.K. government's support for the “hard” Brexit option was announced in September 2016. Study 2 examined the same predictions as Study 1 and sought to clarify that collective narcissism, rather than national attachment, would predict the Brexit vote and support for the referendum's outcome via the perceived threat of immigrants.

### Participants

The same criteria for the sample size were chosen as in Study 1 but the platform did not effectively filter out participants who did not vote in the referendum. The requested sample was 251 participants but 25 participants reported not having voted in the E.U. referendum and their data were excluded from analyses. Out of the 226 participants who reported voting in the E.U. referendum, 161 reported voting to remain in the E.U. The mean age was 34.37 (*SD* = 11.89), 125 were women. One hundred and thirty-four participants identified as English, 20 as Welsh, 7 as Scottish, 2 as Irish, 57 as British, 5 as U.K., and 1 as Other.

### Procedure

The procedure was the same as in Study 1. Participants in Study 1 could not take part in Study 2 (were defined as non-eligible in Prolific Academic).

#### Measurements

Study 2 controlled for participants' age, gender (0 = “*Male,”* 1 = “*Female”*), education, ethnicity (0 = “*Black”* or “*Asian”* or “*Other,”* 1 = “*White”*), and reported national group (0 = “*English”* or “*Welsh”* or “*Scottish”* or “*Irish*,” 1 = “*British”* or “*U.K.”*). As in Study 1 all measures were randomly presented. ***Collective narcissism***, (α = 0.88), ***right-wing authoritarianism*** (α = 0.80), ***social dominance orientation*** (α = 0.82), and the ***perceived threat of immigrants in the U.K.*** (α = 0.97) and a ***support for the referendum's outcome*** (α = 93) were measured as in Study 1.

##### National attachment

National attachment was assessed by the ingroup satisfaction subscale of the Ingroup Identity Scale which pertains to positive attachment to a national group (α = 0.91; Leach et al., 2008; e.g., “*I am glad to be a member of my national group”; “I think that members of my national group have a lot to be proud of”; “It is pleasant to be member of my national group”;* and “*Being a member of my national group gives me a good feeling”*). Items were answered on scales from “1” = “*I strongly disagree”* to “7” = “*I strongly agree.”*

### Results

#### Predictors of prejudice and the referendum vote via the perceived threat of immigrants

All continuous variables were positively correlated (Table 4). As in Study 1, to test whether collective narcissism, right wing authoritarianism and social dominance orientation independently predict the Brexit vote via the perceived threat of immigrants, multiple mediation analyses in the multiple regression context with the self-reported vote in the E.U. referendum as a binary outcome variable were performed three times for each predictor separately, and the other predictors as covariates. Next, national attachment and demographic covariates were entered into the equation. Finally, as in Study 2, we entered national attachment as a predictor instead of collective narcissism and run the analyses without and with collective narcissism as a covariate.

**Table 4:** Descriptive statistics and correlations for the measurements of Study 2, *N* = 226.

**Variables**	**Mean**	***SD***	**Correlations**				
			**1**.	**2**.	**3**.	**4**.	**5**.
1. Collective narcissism	2.72	1.11	—				
2. National attachement	4.72	1.24	0.47^***^	—			
3. Social dominance orientation	2.43	1.07	0.44^***^	0.21^***^	—		
4. Right-wing authoritarianism	3.08	0.99	0.50^***^	0.35^***^	0.50^***^	—	
5. Perceived threat of immigrants	2.78	1.48	0.57**	0.35^***^	0.49^***^	0.50^***^	—
6. Support for outcome	3.79	1.21	0.37^***^	0.25^***^	0.36^***^	0.37^***^	0.63^***^

*** *p < 0.001*.

**Figure 2:**
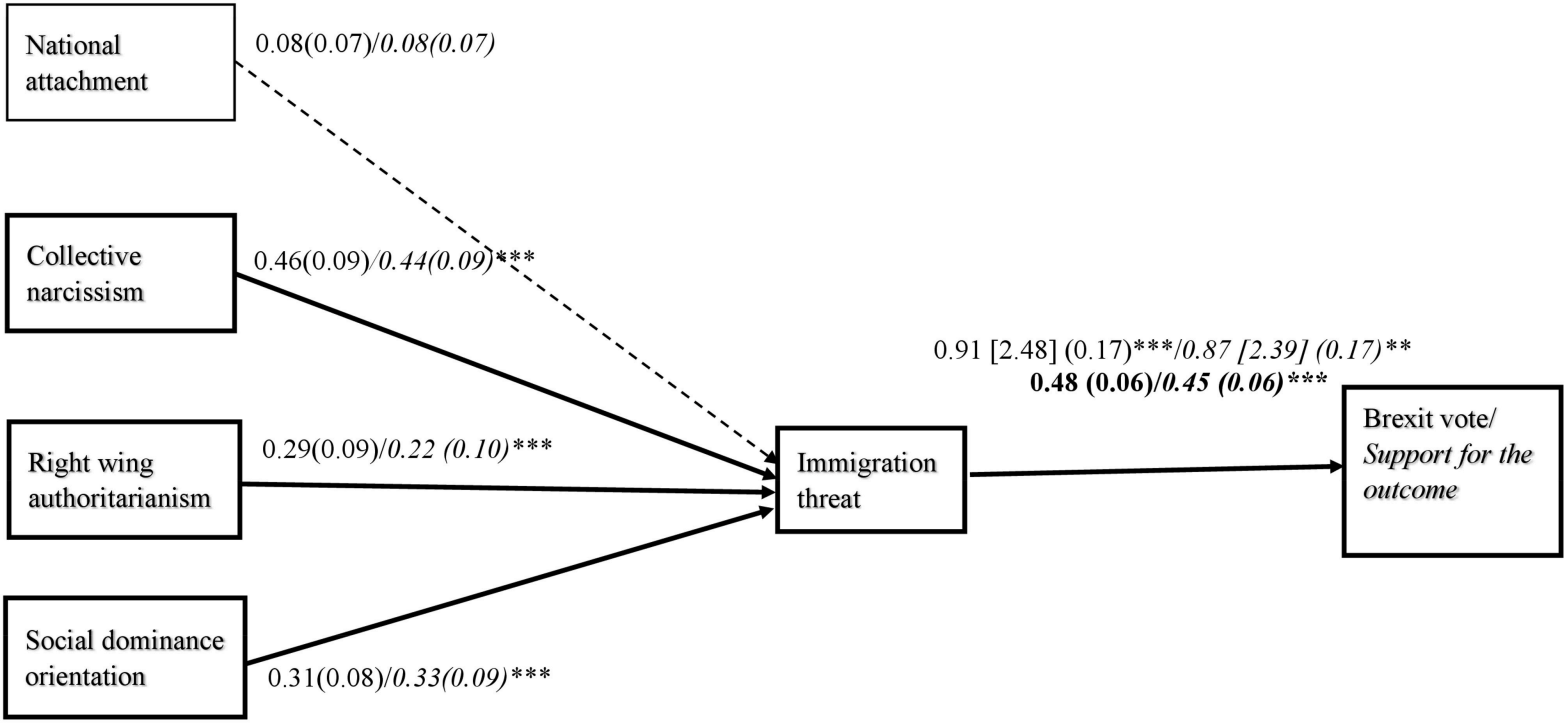
Relationship between variables in Study 2. Coefficients for the binary outcome variable represent log odds [odd ratios] and standard errors are in parentheses. Coefficients for the continuous variables represent unstandardized regression weights. Numerals in italic font correspond to analyses with covariates (national identification, age, gender, education, ethnicity, and indicated national group). Numerals in bold font correspond to unstandardized regression weights and standard error in analyses predicting the continuous support for the outcome of the referendum. ^***^p < 0.001; ^**^p < 0.01.

The analyses for the first model indicated that the whole model was significant, *R*_*CS*_^2^ = 0.28(0.40 Nagelkerke), $\chi_{\left(1\right)}^{2}$ = 196.62, *p* < 0.001. The analyses indicated that collective narcissism, right wing authoritarianism and social dominance were independently related the Brexit vote via the perceived threat of immigrants (Table 2, Figure 2). Overall the pattern of relationships remained unchanged after the covariates were entered to the equation, with the exception of right wing authoritarianism. The analyses with national attachment as a predictor instead of collective narcissism produced a significant model, *R*_*CS*_^2^ = 0.27(0.40 Nagelkerke), $\chi_{\left(1\right)}^{2}$ = 196.92, *p* < 0.001. The direct effect of national attachment on the referendum vote was not significant, *b* = 0.03, SE = 0.17, 95%CI [−0.30; 0.37], *z* = 0.20, *p* = 0.84. However, the indirect effect via the perceived threat of immigrants was positive and significant, IE = 0.20, SE = 0.08, 95%CI [0.07; 0.39], *z* = 2.75, *p* = 0.006. When collective narcissism was entered as a covariate this effect became non-significant, IE = 0.08, SE = 0.07, 95%CI [−0.04; 0.21], *z* = 1.16, *p* = 0.24. This pattern remained unchanged after the demographic covariates were also entered into the equation (Table 2).

#### Predictors of prejudice and support for the referendum's outcome via the perceived threat of immigrants

Next, we performed the same multiple mediation analysis using support for the referendum outcome as a continuous outcome variable. Collective narcissism, right wing authoritarianism, and social dominance orientation were entered as predictors, the perceived threat of immigrants as the mediator and a support for the referendum's outcome as the outcome variable. The analyses were performed three times using PROCESS (Model 4, Hayes, 2013). Next, the analyses were performed with national attachment and demographic variables as covariates. Finally, the analyses were performed with national attachment as a predictor instead of collective narcissism, first without and then with collective narcissism as a covariate.

The first set of analyses produced a significant model, *R*^2^ = 0.40, *F*_(4, 221)_ = 37.83, *p* < 0.001. The analyses indicated significant, independent, indirect effects of collective narcissism, right wing authoritarianism, and social dominance orientation on support for the referendum's outcome via the perceived threat of immigrants (Table 2, Figure 2). The analyses with national attachment as a predictor instead of collective narcissism produced a significant model, *R*^2^ = 0.41, *F*_(4, 221)_ = 37.90, *p* < 0.001. The direct effect of national attachment on the referendum vote was not significant, *b* = 0.03, SE = 0.06, 95%CI [−0.08; 0.14], *t* = 0.48, *p* = 0.63. However, the indirect effect via the perceived threat of immigrants was positive and significant, IE = 0.10, SE = 0.04, 95%CI [0.04; 0.18], *z* = 2.99, *p* = 0.003. When collective narcissism was entered as a covariate this effect became non-significant, IE = 0.04, SE = 0.03, 95%CI [−0.02; 0.10], *z* = 1.19, *p* = 0.23. This pattern remained unchanged after the demographic covariates were entered into the equation (Table 2).

#### Relative importance of all predictors of prejudice for the perceived threat of immigrants and support for the referendum's outcome

Again, the relative importance indices analysis indicated an independent and closely comparable contribution of collective narcissism, right wing authoritarianism, and social dominance orientation to explaining the variance in the perceived threat of immigrants and a positive attitude toward the referendum's outcome. The relative role of national attachment was negligible (Table 5).

**Table 5:** Comparison of relative importance of all variables in explaining variance in support for the referendum's outcome, Study 2, *N* = 226.

**Variables**	**VIF**	**Study 2 (*threat of immigrants*, Overall *R*^2^ = 0.43)**				**Study 2 (*support for the outcome*, Overall *R*^2^ = 0.21)**			
		**Regression weights**	**Dominance weights**	**Relative importance weights**	**Incremental *R*^2^**	**Regression weights**	**Dominance weights**	**Relative importance weights**	**Incremental *R*^2^**
Collective narcissism	1.66	0.34	0.17	0.16	0.07	0.17	0.06	0.06	0.02
National identification/attachment	1.32	0.08	0.04	0.05	0.004	0.07	0.02	0.02	0.004
Social dominance orientation	1.43	0.23	0.11	0.11	0.04	0.19	0.06	0.07	0.02
Right wing authoritarianism	1.57	0.19	0.11	0.10	0.02	0.17	0.06	0.06	0.02

## Discussion

The outcome of the “European referendum” on June 23, 2016 was largely unexpected. It has been explained by voter's age (i.e., older voters), their economic situation (i.e., poorer voters), and education (less educated voters especially in high-skilled areas) suggesting that the Brexit vote reflected a broader societal division between those who embraced modern globalized economy and groups that were “left behind” by the economic growth in the U.K. (Goodwin and Health, 2016). The analyses considering individual difference predictors of the Brexit vote pointed to voters' cost-benefit and risk calculations, susceptibility to influence of political elites and low feelings of attachment to the wider international community (Clarke et al., 2016). While these explanations provided important insights into factors that played a role in the Brexit vote, the role of xenophobia, the fear of foreigners, was often alluded to, but never systematically examined, as a psychological factor behind the Brexit vote. However, before the referendum, the Leave campaign mobilized anxiety over the national prosperity and sovereignty being threatened by foreign immigration to the U.K. The Leave campaign framed leaving the E.U. as resurrecting the national greatness undermined by foreign forces: the E.U. and immigrants (Clarke et al., 2016). The present results suggest that this framing might have addressed the concerns of people already prone to prejudice against foreigners.

All three robust predictors of prejudice were related to the Brexit vote and the support for the referendum's outcome via the perceived threat of immigrants: collective narcissism, right wing authoritarianism, and social dominance orientation. This suggests that, the Brexit vote was motivated by different concerns instigating prejudice: the collective narcissistic concern regarding the recognition of the national group's uniqueness, authoritarian concern regarding protection of the normative status quo, and the concern regarding protection of the national group's elevated international status, relevant to people high in social dominance orientation. The individual difference predictors of xenophobia explained the variance in the Brexit vote and the support for the referendum's outcome over and above demographic predictors such as age, ethnicity or education (frequently commented on factors that predicted the referendum vote, Waugh, 2017).

Collective narcissism was an independent and at least equally strong (if not stronger) predictor of the perceived threat of immigrants and support for the referendum's outcome as the other robust psychological predictors of xenophobia: right wing authoritarianism and social dominance orientation. The results of the dominance and relative importance analyses suggested that the contribution of the three variables to explaining the variance in the perceived threat of immigrants and the support for the referendum's outcome was independent and comparable in size. These results corroborate previous findings indicating that collective narcissism, right wing authoritarianism, and social dominance orientation explain negativity toward other groups independently and for different reasons (Golec de Zavala et al., 2009, 2013b, 2016). Thus, the present results indicated that collective narcissism should be considered as an equally important and stable individual difference predictor of bigotry, as right wing authoritarianism and social dominance orientation. The present results also suggested that collective narcissism should be considered as a predictor of political behavior at least as important as right wing authoritarianism and social dominance orientation. Previous studies that considered this variable in the context of voting indicated that it predicted voting for Donald Trump in the Presidential election in the U.S. over and above right wing authoritarianism. This suggest that predictor of prejudice play a role in another voting for a candidate whose political campaign stirred anti-immigrant sentiments.

To the best of our knowledge, the present results are the first to demonstrate that all three predictors of xenophobia are related to the rejection of the superordinate group that would undermine the strength of the boundaries between the national ingroup and outgroups, i.e., citizens of the E.U. These results corroborate previous findings pointing to the limited enthusiasm for the E.U. among those who identify strongly as British (e.g., Cinnirella, 1997; Cinnirella and Hamilton, 2007). However, the current results qualify previous findings indicating that it is collective narcissism that predicts lower support for the E.U., rather than national identification or national attachment. National identification (how important it is for people to be members of the national group) and national attachment (how attached and positive people feel about their national groups) were not related to rejection of immigrants, the Brexit vote or the support for the referendum's outcome after their overlap with other three predictors was controlled for. The results are especially interesting with reference to national attachment, which predicted the perceived threat of immigrants when analyses did not control for its overlap with collective narcissism. This suggests that national attachment may explain the perceived threat of immigrants but only in as much as it is related to collective narcissism. These results corroborate previous findings suggesting that collective narcissism is a distinct form of ingroup positivity that systematically predicts intergroup hostility in the context of intergroup threat (Golec de Zavala, in press).

Specifically, previous studies showed that in contrast to other forms of ingroup positivity (such as collective self-esteem, positive ingroup identification, or constructive patriotism) collective narcissism was reliably related to negative attitudes and hostility toward outgroups. In addition, controlling for the overlap between collective narcissism and ingroup attachment allowed the latter to emerge as a predictor of outgroup tolerance (Golec de Zavala et al., 2013a). In previous studies, non-narcissistic (statistically freed from the overlap with collective narcissism) ingroup attachment did not predict hypersensitivity to intergroup threat (Golec de Zavala et al., 2016) and was not related to conspiracy beliefs about Jews or siege mentality (Golec de Zavala and Cichocka, 2012). The present results go beyond such findings, indicating that only collective narcissism, but not national identification or national attachment, was related to rejection of immigrants to the U.K. and support for the Brexit vote.

In the present research the common variance was removed from all relevant concepts: collective narcissism, national identification, and national attachment but also social dominance orientation and right wing authoritarianism. Partialling out the common variance can create problems with interpreting the residual variables (Lynam et al., 2006). In order to prevent this problem we followed the steps prescribed by the above mentioned authors. We used validated and highly reliable scales (internal consistencies ranged from 0.78 to 0.97) and analyzed mediation relationships in our data. We used variables that are theoretically differentiable and generally well-understood as distinct constructs. Right wing authoritarianism and social dominance orientation have been differentiated as submissive vs. dominant facets of authoritarianism. They were related to different outcomes and have different antecedents (Duckitt, 2006; Sibley et al., 2007a,b). They were also clearly differentiated from national attitudes (Osborn et al., 2017). Similarly, previous research showed that feeling proud and satisfied to be a member of a valuable group is psychologically distinct from collective narcissism. While collective narcissism and non-contingent in-group positivity may quite often coexist, they seem to refer to different psychological realities (Golec de Zavala, in press). Non-narcissistic ingroup positivity was related to high self-esteem, whereas collective narcissism was associated with low self-esteem via vulnerable narcissism (Golec de Zavala, in press). Ingroup positivity, with collective narcissism partialled out, can be interpreted as a confident positive evaluation of the ingroup, independent of external recognition and resilient to threats and criticism. Collective narcissism, with ingroup positivity partialled out, can be interpreted as group-based entitlement without the comfort of the sense of belonging to a valuable group (Golec de Zavala, in press).

Our interpretation of the present findings can be based on the raw variables because partialling their common variance did not increase or change the direction of their zero-sum associations with the mediators and outcomes (except of the already discussed case of national attachment). Finally, we used the dominance and relative importance analyses to determine the relative contribution of correlated predictors to explaining the variance in the mediator and the outcome variables. Such an approach increases our confidence that our results present distinct contributions of the predictors of prejudice to explaining the variance in the perceived threat of immigrants and support for the Brexit vote.

## Limitations and future directions

Although, the present studies provide novel insights into the individual level variables related to support for the E.U. in the UK, they are not without limitations. Although, the results were obtained on sample that are large enough to reveal stable correlations, those results are not representative. The sampling was opportunistic and resulted in a sample with majority of voters who chose the “Remain” option. However, the fact that the results were remarkably consistent across two different samples of British adults increases our confidence in generalizability of our findings. Another limitation may be the fact that the mediator and the continuous variable were quite strongly correlated. However, we performed similar mediation analyses with a binary outcome variable (i.e., participants vote in the referendum). The results of those analyses closely matched the results obtained with the continuous outcome variable i.e., the support for the referendum's outcome. Nevertheless, the results are correlational and cross-sectional. They do not allow for firm causal inferences or firm inferences regarding directionality of the effects. However, we provide theoretical reasons to justify that it is likely that psychological predictors of prejudice should be related to the rejection of immigrants and political choices rather than the other way around. In addition, most research in the social sciences confirm the direction of causality assumed in the tested model, suggesting that broader ideological orientations and basic ingroup identification (such as right wing authoritarianism, social dominance orientation, and collective narcissism) constrain specific beliefs and actions, such as the perceived threat of immigrants, the vote in the referendum and support for the referendum's outcome (rather than vice versa; see e.g., De Figueiredo and Elkins, 2003; Cohrs et al., 2005; Duckitt, 2006).

Arguably, different causation could also be plausible. Collective narcissism, right wing authoritarianism, and social dominance orientation might have predicted the Brexit vote and support for the referendum's outcome that strengthen the perceived rejection of immigrants. We tested such alternative mediation models (with the support for the referendum's outcome entered as a continuous mediator and the perceived threat of immigrants as the outcome variable). The fit to the data was worse in comparisons to the models we proposed and tested. In addition, the alternative models showed inconsistent relationships in Study 1 and 2. In Study 1, the indirect effects via the support for the referendum's outcome to the perceived threat of immigrants were significant for collective narcissism and right wing authoritarianism but not for social dominance orientation. Those effects were weaker than the effects in the models we proposed. In Study 2, the reverse indirect effect was not significant for collective narcissism. Nevertheless, the cross-sectional nature of the present data is not optimal for testing mediation models and future studies would do well to examine collective narcissism, right wing authoritarianism, and social dominance orientation as predictors of political behavior in panel designs assessing predictors and mediators at different times.

## Conclusions

We argue that our analyses offer a novel perspective on the Brexit vote and the support for the referendum's outcome. They indicate that psychological predictors of xenophobia were related to the rejection of the U.K.'s membership in the European Union. Understanding whether prejudice motivated the Brexit vote or the Brexit vote legitimized and increased prejudice may be of lesser importance than understanding that individual predictors of prejudice are related to political choices that undermine diversity and harmonious intergroup relations. The mobilization of xenophobic sentiments around Brexit also suggests that, at least to some extent, this political choice was motivated by affect rather than rational consideration of collective costs and benefits. The present results suggest that at least three categories of concerns that go beyond cost-benefit and risk calculations are relevant to the Brexit process: undermined national uniqueness (concern associated with collective narcissism), the threatened traditional status quo (concern associated with right wing authoritarianism), and threatened international status (concern associated with social dominance orientation). Whether those concerns should be given precedence over the country's welfare and internal stability is the subject of ongoing political debates.

## Ethics statement

The studies presented in this manuscripts were approved by the Ethics Committee at the Department of Psychology, Goldsmiths, University of London. Participants were informed about the procedure of the topic study of the study beforehand. They were given the option not to answer the survey questions and withdraw their participation or their data at any point without giving reasons. Participants were informed that their data will be used for scientific purposes only and that no individualized statements can be made based on the data analyses. They were informed their answers are anonymous and cannot be linked to any personalized information. Participants were debriefed as to study hypotheses in the end of the study.

## Author contributions

AG contributed the research idea and design of the studies. She helped analyze the data and oversaw the preparation of the manuscript. She was responsible for analyzing the data and presentation of the theory providing the major draft of the Introduction, Results, and Discussion sections. RG contributed the data analyses and presentation. She helped preparation of the manuscript overseeing especially the Method and Results sections. CS oversaw the process of data collection, helped with data analysis and presentation, and edited the final versions of the manuscript.

### Conflict of interest statement

The authors declare that the research was conducted in the absence of any commercial or financial relationships that could be construed as a potential conflict of interest.

## References

[B1] Aichholzer J. Zandonella M. (2016). Psychological bases of support for radical right parties. Pers. Individ. Dif. 96, 185–190. 10.1016/j.paid.2016.02.072

[B2] Altemeyer B. (1988). Enemies of Freedom: Understanding Right-wing Authoritarianism. San Francisco, CA: Jossey-Bass.

[B3] Asbrock F. Sibley C. G. Duckitt J. (2010). Right-wing authoritarianism and social dominance orientation and the dimensions of generalized prejudice: a longitudinal test. Eur. J. Pers. 24, 324–340. 10.1002/per.746

[B4] Braun M. T. Oswald F. L. (2011). Exploring regression analysis: a tool for selecting models and determining predictor importance. Behav. Res. Methods 43, 331–339. 10.3758/s13428-010-0046-8 21298571

[B5] Cai H. Gries P. (2013). National narcissism: internal dimensions and international correlates. PsyCh J. 2, 122–132. 10.1002/pchj.26 26271182

[B6] Cameron J. (2004). A three-component model of social identification. Self Ident. 3, 239–262. 10.1080/13576500444000047

[B7] Carrey S. (2002). Undivided loyalties: is national identity an obstacle to European integration? Eur. Union Politics 3, 387–413. 10.1177/1465116502003004001

[B8] Choma B. L. Hanoch Y. (2017). Cognitive ability and authoritarianism: understanding support for Trump and Clinton. Pers. Individ. Dif. 106, 287–291. 10.1016/j.paid.2016.10.054

[B9] Cichocka A. (2016). Understanding defensive and secure in-group positivity: the role of collective narcissism. Eur. Rev. Soc. Psychol. 27, 283–317. 10.1080/10463283.2016.1252530

[B10] Cinnirella M. (1997). Towards a European identity? Interactions between the national and European social identities manifested by university students in Britain and Italy. Br. J. Soc. Psychol. 36, 19–31. 10.1111/j.2044-8309.1997.tb01116.x

[B11] Cinnirella M. Hamilton S. (2007). Are all Britons reluctant Europeans? Exploring European identity and attitudes to Europe among British citizens of South Asian ethnicity. Ethn. Racial Stud. 30, 481–501. 10.1080/01419870701217530

[B12] Clarke H. Goodwin M. Whiteley P. (2016). Why Britain voted for Brexit: an individual-level analysis of the 2016 referendum vote, in Paper Presented at EPOP Conference, Sept. 10 (Kent).

[B13] Cohen M. J. Smith A. E. (2016). Do authoritarians vote for authoritarians? Evidence from America Latina. Res. Polit. 3, 1–8. 10.1177/2053168016684066

[B14] Cohrs J. C. Moschner B. Maes J. Kielmann S. (2005). The motivational bases of right-wing authoritarianism and social dominance orientation: relations to values and attitudes in the aftermath of September 11, 2001. Pers. Soc. Psychol. Bull. 31, 1425–1434. 10.1177/0146167205275614 16143673

[B15] Cornelis I. Van Hiel A. (2015). Extreme-right voting in Western Europe: the role of social-cultural and antiegalitarian attitudes. Politic. Psychol. 36, 749–760. 10.1111/pops.12187

[B16] Cottrell C. A. Neuberg S. L. (2005). Different emotional reactions to different groups: a sociofunctional threat-based approach to “prejudice”. J. Pers. Soc. Psychol. 88, 770–789. 10.1037/0022-3514.88.5.770 15898874

[B17] Crawford J. T. Brady J. L. Pilanski J. M. Erny H. (2013). Differential effects of right-wing authoritarianism and social dominance orientation on political candidate support: the moderating role of message framing. J. Soc. Polit. Psychol. 1, 5–28. 10.5964/jspp.v1i1.170

[B18] Crowson H. M. Brandes J. A. (2017). Differentiating between Donald Trump and Hilary Clinton voters using facets of right-wing authoritarianism and social-dominance orientation. Psychol. Rep. 120, 364–373. 10.1177/0033294117697089 28558614

[B19] De Figueiredo R. J. Elkins Z. (2003). Are patriots bigots? An inquiry into the vices of in-group pride. Am. J. Polit. Sci. 47, 171–188. 10.1111/1540-5907.00012

[B20] Dru V. (2007). Authoritarianism, social dominance orientation and prejudice: effects of various self-categorization conditions. J. Exp. Soc. Psychol. 43, 877–883. 10.1016/j.jesp.2006.10.008

[B21] Duckitt J. (2006). Differential effects of right wing authoritarianism and social dominance orientation on outgroup attitudes and their mediation by threat from and competitiveness to outgroups. Pers. Soc. Psychol. Bull. 32, 684–696. 10.1177/0146167205284282 16702160

[B22] Duckitt J. Bizumic B. Krauss S. W. Heled E. (2010). A tripartite approach to right-wing authoritarianism: the authoritarianism-conservatism-traditionalism model. Polit. Psychol. 31, 685–715. 10.1111/j.1467-9221.2010.00781.x

[B23] Duckitt J. Sibley C. G. (2007). Right wing authoritarianism, social dominance orientation and the dimensions of generalized prejudice. Eur. J. Pers. 21, 113–130. 10.1002/per.614

[B24] Ekehammar B. Akrami N. Gylje M. Zakrisson I. (2004). What matters most to prejudice: big five personality, social dominance orientation, or right-wing authoritarianism? Eur. J. Personal. 18, 463–482. 10.1002/per.526

[B25] Elliott M. (2016). Brexit “Vote Leave to Take Control”? Sovereignty and the Brexit Debate. Available online at: https://publiclawforeveryone.com/2016/06/23/vote-leave-take-control-sovereignty-and-the-brexit-debate/ (Accessed March 21, 2017).

[B26] Esses V. M. Medianu S. Lawson A. S. (2013). Uncertainty, threat, and the role of the media in promoting the dehumanization of immigrants and refugees. J. Soc. Issues 69, 518–536. 10.1111/josi.12027

[B27] Federico C. Golec de Zavala A. (in press). Collective narcissism in the 2016 Presidential election*. Public Opin. Q*. Available online at: https://www.washingtonpost.com/news/monkey-cage/wp/2017/03/17/collective-narcissism-explains-at-least-some-of-president-trumps-support/?utm_term=.515f97b61a3c. (Retrieved on: 10 November, 2017).

[B28] Forster K. (2016, October 13). Hate crimes Soared by 41% After Brexit Vote, Official Figures Reveal. Independent. Available online at: http://www.independent.co.uk/news/uk/crime/brexit-hate-crimes-racism-eu-referendum-vote-attacks-increase-police-figures-official-a7358866.html.

[B29] Golec de Zavala A. (2017). Collective narcissism: antecedents and consequences of exaggeration of the in-group image, in The Handbook of Trait Narcissism: Key Advances, Research Methods, and Controversies, eds Hermann A. Brunell A. Foster J. (New York, NY: Springer).

[B30] Golec de Zavala A. Cichocka A. Eidelson R. Jayawickreme N. (2009). Collective narcissism and its social consequences. J. Pers. Soc. Psychol. 97, 1074–1096. 10.1037/a0016904 19968420

[B31] Golec de Zavala A. Cichocka A. Bilewicz M. (2013a). The paradox of in-group love: differentiating collective narcissism advances understanding of the relationship between in-group and out-group attitudes. J. Pers. 81, 16–28. 10.1111/j.1467-6494.2012.00779.x 22329421

[B32] Golec de Zavala A. Cichocka A. Iskra-Golec I. (2013b). Collective narcissism moderates the effect of in-group image threat on intergroup hostility. J. Pers. Soc. Psychol. 104, 1019–1039. 10.1037/a0032215 23586408

[B33] Golec de Zavala A. Cichocka A. K. (2012). Collective narcissism and anti-Semitism in Poland. Group Process. Intergroup Relat. 15, 213–229. 10.1177/1368430211420891

[B34] Golec de Zavala A. Peker M. Guerra R. Baran T. (2016). Collective narcissism predicts hypersensitivity to in-group insult and direct and indirect retaliatory intergroup hostility. Eur. J. Pers. 30, 532–551. 10.1002/per.2067

[B35] Goodwin M. Health O. (2016, August 31). Brexit Vote Explained: Poverty, Low Skills Lack of Opportunities. Available online at: https://www.jrf.org.uk/report/brexit-vote-explained-poverty-low-skills-and-lack-opportunities?gclid=CjwKCAjwpfzOBRA5EiwAU0ccNw4DjkpPU03nS7-Y4zdDfW86S8gHjcDw1a5Kpi0WqcJWxyfVrU7Z2hoCYjsQAvD_BwE

[B36] Gries P. Sanders M. A. Stroup D. R. Cai H. (2008). Hollywood in China: how American popular culture shapes Chinese views of the “beautiful imperialist” – An experimental analysis. China Q. 224, 1070–1082. 10.1017/S0305741015000831

[B37] Hayes A. F. (2013). Introduction to Mediation, Moderation, and Conditional Process Analysis: A Regression-Based Approach. New York, NY: Guilford Press.

[B38] Herrmann R. K. Isernia P. Segatti P. (2009). Attachment to the nation and international relations: dimensions of identity and their relationship to war and peace. Polit. Psychol. 30, 721–754. 10.1111/j.1467-9221.2009.00723.x

[B39] Hibbing J. R. Smith K. B. Alford J. R. (2014). Differences in negativity bias underlie variations in political ideology. Behav. Brain Sci. 37, 297–307. 10.1017/S0140525X13001192 24970428

[B40] Ho A. K. Sidanius J. Pratto F. Levin S. Thomsen L. Kteily N.. (2012). Social dominance orientation: revisiting the structure and function of a variable predicting social and political attitudes. Pers. Soc. Psychol. Bull. 38, 583–606. 10.1177/0146167211432765 22215697

[B41] Hobolt S. B. (2016). The Brexit vote: a divided nation, a divided continent. J. Eur. Public Policy 23, 1259–1277. 10.1080/13501763.2016.1225785

[B42] Jost J. T. Glaser J. Krugalanski A. W. Sulloway F. J. (2003). Political conservatism as motivated social cognition. Psychol. Bull. 129, 339–375. 10.1037/0033-2909.129.3.339 12784934

[B43] Kaufman (2016). It is Not the Economy, Stupid: Brexit As a Story of Personal Values. Available online at: http://blogs.lse.ac.uk/politicsandpolicy/personal-values-brexit-vote (Accessed March 21, 2017).

[B44] Kosterman R. Feshbach S. (1989). Toward a measure of patriotic and nationalistic attitudes. Politic. Psychol. 10, 257–274.

[B45] Kteily N. S. Sheehy-Skeffington J. Ho A. K. (2017). Hierarchy in the eye of the beholder: (Anti-)egalitarianism shapes perceived levels of social inequality. J. Pers. Soc. Psychol. 112, 136–159. 10.1037/pspp0000097 27124377

[B46] Lavine H. Burgess D. Snyder M. Transue J. van Sulli J. L. Haney B. (1999). Threat, authoritarianism, and voting: an investigation of personality and persuasion. Pers. Soc. Bull. 25, 337–347. 10.1177/0146167299025003006

[B47] Leach C. W. Van Zomeren M. Zebel S. Vliek M. L. Pennekamp S. F. Doosje B.. (2008). Group-level self-definition and self-investment: a hierarchical (multicomponent) model of in-group identification. J. Pers. Soc. Psychol. 95:144. 10.1037/0022-3514.95.1.144 18605857

[B48] Lynam D. R. Hoyle R. H. Newman J. P. (2006). The perils of partialling: cautionary tales from aggression and psychopathy. Assessment 13, 328–341. 10.1177/1073191106290562 16880283 PMC3152746

[B49] Lyons P. A. Coursey L. E. Kenworthy J. B. (2013). National identity and group narcissism as predictors of intergroup attitudes toward undocumented Latino immigrants in the United States. Hisp. J. Behav. Sci. 35, 323–335. 10.1177/0739986313488090

[B50] MacWilliams M. (2016). Who decides when the party doesn't? Authoritarian voters and the rise of Donald Trump. Polit. Sci. Polit. 49, 716–721. 10.1017/S1049096516001463

[B51] Mummendey A. Klink A. Brown R. (2001). Nationalism and patriotism: national identification and out-group rejection. Br. J. Soc. Psychol. 40, 159–172. 10.1348/014466601164740 11446222

[B52] Murray K. E. Marx D. M. (2013). Attitudes toward unauthorized immigrants, authorized immigrants, and refugees. Cultur. Divers. Ethnic Minor. Psychol. 19:332. 10.1037/a0030812 23148903

[B53] Osborn D. Milojev P. Sibley C. G. (2017). Authoritarianism and national identity: examining the longitudinal effects of SDO and RWA on nationalism and patriotism. Pers. Soc. Psychol. Bull. 43, 1086–1099. 10.1177/0146167217704196 28903711

[B54] Pehrson S. Brown R. Zagefka H. (2009). When does national identification lead to the rejection of immigrants? Cross-sectional and longitudinal evidence for the role of essentialist ingroup definitions. Br. J. Soc. Psychol. 48, 61–76. 10.1348/014466608X288827 18302807

[B55] Pratto F. Çidam A. Stewart A. L. Zeineddine F. B. Aranda M. Aiello A. (2013). Social dominance in context and in individuals: contextual moderation of robust effects of social dominance orientation in 15 languages and 20 countries. Soc. Psychol. Personal. Sci. 4, 587–599. 10.1177/1948550612473663

[B56] Pratto F. Sidanius J. Stallworth L. M. Malle B. F. (1994). Social dominance orientation: a personality variable predicting social and political attitudes. J. Pers. Soc. Psychol. 67, 741–763. 10.1037/0022-3514.67.4.741

[B57] Roccas S. Klar Y. Liviatan I. (2006). The paradox of group-based guilt: modes of national identification, conflict vehemence, and reactions to the in-group's moral violations. J. Pers. Soc. Psychol. 91, 698–711. 10.1037/0022-3514.91.4.698 17014294

[B58] Rubin M. Hewstone M. (1998). Social identity theory's self-esteem hypothesis: a review and some suggestions for clarification. Pers. Soc. Psychol. Rev. 2, 40–62. 10.1207/s15327957pspr0201_3 15647150

[B59] Schatz R. T. Staub E. Lavine H. (1999). On the varieties of national attachment: blind versus constructive patriotism. Polit. Psychol. 20, 151–174. 10.1111/0162-895X.00140

[B60] Schönbrodt F. D. Perugini M. (2013). At what sample size do correlations stabilize?. J. Res. Pers. 47, 609–612. 10.1016/j.jrp.2013.05.009

[B61] Sekerdej M. Roccas S. (2016). Love versus loving criticism: disentangling conventional and constructive patriotism. Br. J. Soc. Psychol. 55, 499–521. 10.1111/bjso.12142 27009883

[B62] Sibley C. G. Robertson A. Wilson M. S. (2007a). Social dominance orientation and right-wing authoritarianism: additive and interactive effects. Polit. Psychol. 27, 755–768. 10.1111/j.1467-9221.2006.00531.x

[B63] Sibley C. G. Wilson M. S. Duckitt J. (2007b). Effects of dangerous and competitive worldviews on right-wing authoritarianism and social dominance orientation over a five-month period. Polit. Psychol. 28, 357–371. 10.1111/j.1467-9221.2007.00572.x

[B64] Sidanius J. Levin S. Liu J. Pratto F. (2000). Social dominance orientation, anti-egalitarianism and the political psychology of gender: an extension and cross-cultural replication. Eur. J. Soc. Psychol. 30, 41–67. 10.1002/(SICI)1099-0992(200001/02)30:1<41::AID-EJSP976>3.0.CO;2-O

[B65] Smith E. R. Mackie D. M. (2015). Dynamics of group-based emotions: insights from intergroup emotions theory. Emot. Rev. 7, 39–354. 10.1177/1754073915590614

[B66] Waugh P. (2017). Revealed: Britain's Deep Divisions in the Brexit Vote, with Education, Race and Age Key Factors. Available online at: http://www.huffingtonpost.co.uk/entry/brexit-new-eur-referendum-bbc-analysis-age-race-educational-qualification_uk_58986ffce4b0a1dcbd02faf7 (Accessed June 30, 2017).

[B67] Whitley B. E. Jr. (1999). Right-wing authoritarianism, social dominance orientation, and prejudice. J. Pers. Soc. Psychol. 77:126 10.1037/0022-3514.77.1.126

[B68] Zakrisson I. (2005). Construction of a short version of the Right-Wing Authoritarianism (RWA) scale. Pers. Individ. Dif. 39, 863–872. 10.1016/j.paid.2005.02.026

